# Analysis of the anatomic relationship of the infraorbital canal with the roots of the maxillary fourth premolar tooth in the three different skull types: Mesocephalic, brachycephalic, and dolichocephalic, using cone beam computed tomography

**DOI:** 10.3389/fvets.2022.978400

**Published:** 2022-10-04

**Authors:** Maria E. Littles, Sangeeta Rao, Kristin M. Bannon

**Affiliations:** ^1^Veterinary Dentistry and Oral Surgery of New Mexico, Algodones, NM, United States; ^2^Department of Clinical Sciences, College of Veterinary Medicine and Biomedical Sciences, Colorado State University, Fort Collins, CO, United States

**Keywords:** canine, infraorbital canal, carnassial tooth, computed tomography, maxillary premolar tooth

## Abstract

The objective of this retrospective descriptive study was to describe variations in the anatomic position of the infraorbital canal relative to the mesial and distal roots of the maxillary fourth premolar tooth for the three canine skull types (mesocephalic, brachycephalic, and dolichocephalic) using cone beam computed tomography. The study evaluated the position of the infraorbital canal in 120 canine patients (240 teeth) that presented to a private dentistry referral practice for reasons unrelated to the study. There were 40 patients for each skull type, determined by breed and facial index calculation. A grid system was used to determine the anatomic positions of the infraorbital canal relative to the roots of the maxillary fourth premolar tooth. The infraorbital canal's most frequent position at the mesial roots level for the total population (38.8%) and the mesocephalic skull type (53.8%) was apical to the furcation. For the brachycephalic (40.0%) and the dolichocephalic skull types (40.0%), the most frequent position was partially in the furcation and partially apical to the furcation. The most frequent position of the infraorbital canal at the level of the distal root was apical and palatal to the distal root for the total population (36.7%), the mesocephalic skull type (42.5%), and the brachycephalic skull type (35.0%). For the dolichocephalic skull type, the most frequent location of the infraorbital canal was both directly palatal and partially apical and palatal to the distal root (42.5%). For the brachycephalic skull type, the distal root was most frequently positioned caudal to the maxillary foramen/floor of the orbit (62.5%). Based on these findings, extra care must be taken with the dolichocephalic and the brachycephalic skull types to avoid iatrogenic trauma to the infraorbital canal and the orbit during surgical procedures on the maxillary fourth premolar tooth.

## Introduction

The carnassial teeth, which include the maxillary fourth premolar teeth (P4) and mandibular first molar teeth (M1), are the largest shearing teeth in the oral cavity of dogs (1). Common causes for surgical extraction of the P4 are end-stage periodontal disease, endodontic disease (not amendable to endodontic therapy), crown or crown–root fractures, and tooth resorption (2, 3). The P4 may also require surgical endodontic therapy. Common causes for surgical endodontic therapy are failure of a nonsurgical root canal treatment, anatomical barriers, separation of an endodontic instrument, and cyst formation (4, 5). Knowing the surgical anatomy involving the P4 will lower the risk of iatrogenic complications like hemorrhage, displacement of the root tip, nerve damage, and ocular trauma (6–8). The proximity of the P4 roots to the infraorbital canal (IOC), which contains the infraorbital neurovascular bundle, is of particular importance. In dogs, the position of the IOC has been previously described as being dorsal to the roots of the P4 (9) or passing between the two mesial roots of the P4 (7, 10, 11). Identifying the variations that might exist in the general population and among the different skull types may provide more insight when performing surgical extraction or surgical endodontic therapy on the P4.

To the author's knowledge, no studies describe and compare the position of the IOC relative to the roots of the P4 for the three different skull types: mesocephalic (M), dolichocephalic (D), and brachycephalic (B). This study aimed to accurately describe the anatomic position of the IOC relative to the roots of the P4 using cone beam computed tomography (CBCT) for the three different skull types.

## Materials and methods

### Animals

This retrospective descriptive study involved 120 canine patients evaluated and treated at a private veterinary dentistry referral practice between July 2018 and December 2019. A cone beam computed tomography (CBCT, Xoran VetCat, Xoran Technologies, Ann Arbor, MI) scan was performed on these patients under inhalation general anesthesia for conditions unrelated to the study. Inclusion parameters for this study included: 1 year of age or older, mesocephalic head shape, brachycephalic head shape, dolichocephalic head shape, presence of maxillary fourth premolar teeth bilaterally (with stage 0 or stage 1 periodontal disease, and no evidence of periapical disease, root resorption or root fractures), and no pathology involving the infraorbital canal. Informed consent for the use of the clinical data was obtained by the owners as part of the practice's intake consent form.

### Image acquisition

A veterinary mobile CBCT unit was used to obtain images. Dogs were placed in sternal recumbency for the scans. A standard high-resolution protocol was used to acquire images for the study. The standard field of view was 24 × 14 cm, with a voxel size of 0.3 mm, at the window width of 5,274 Hounsfield units (HU) and window level of 813 HU. Images were viewed and evaluated using the same acquisition software. All images were viewed in the transverse, sagittal, and coronal planes.

### Image evaluation and scoring

Two hundred and forty P4s from a total of 120 dogs were evaluated: 40 dogs (80 P4s) in each skull type group (M, D, and B). The skull type was classified based on breed. The facial index was then calculated for each skull, but this did not affect the skull classification. In the mixed-breed dogs without an obvious breed skull type, the calculated facial index and dominant phenotype determined the skull classification. The sagittal and dorsal planes were used to calculate the facial index. The following equation was used to determine the facial index: Facial index = (facial width × 100)/facial length (9).

The facial width was defined as the widest interzygomatic distance. The facial length was defined as nasion to prosthion (9, 12). The published averages for the facial index for each skull type were 111 for M, 81 for D, and 215 for B and were within the facial index ranges used to categorize mixed-breed dogs (9). The facial index ranges were 104–165 for the mesocephalic skull type, less than 104 for the dolichocephalic skull type, and greater than 165 for the brachycephalic skull type. The facial indices for two individuals in the dolichocephalic skull type group were not calculated because the width of the zygoma and the length from nasion to prosthion could not be measured on the sagittal and dorsal planes. The two individuals were determined to be dolichocephalic based on their breed (borzoi and rough-coated collie). Data about the total population's sex and weight were also collected.

**Figure 1:**
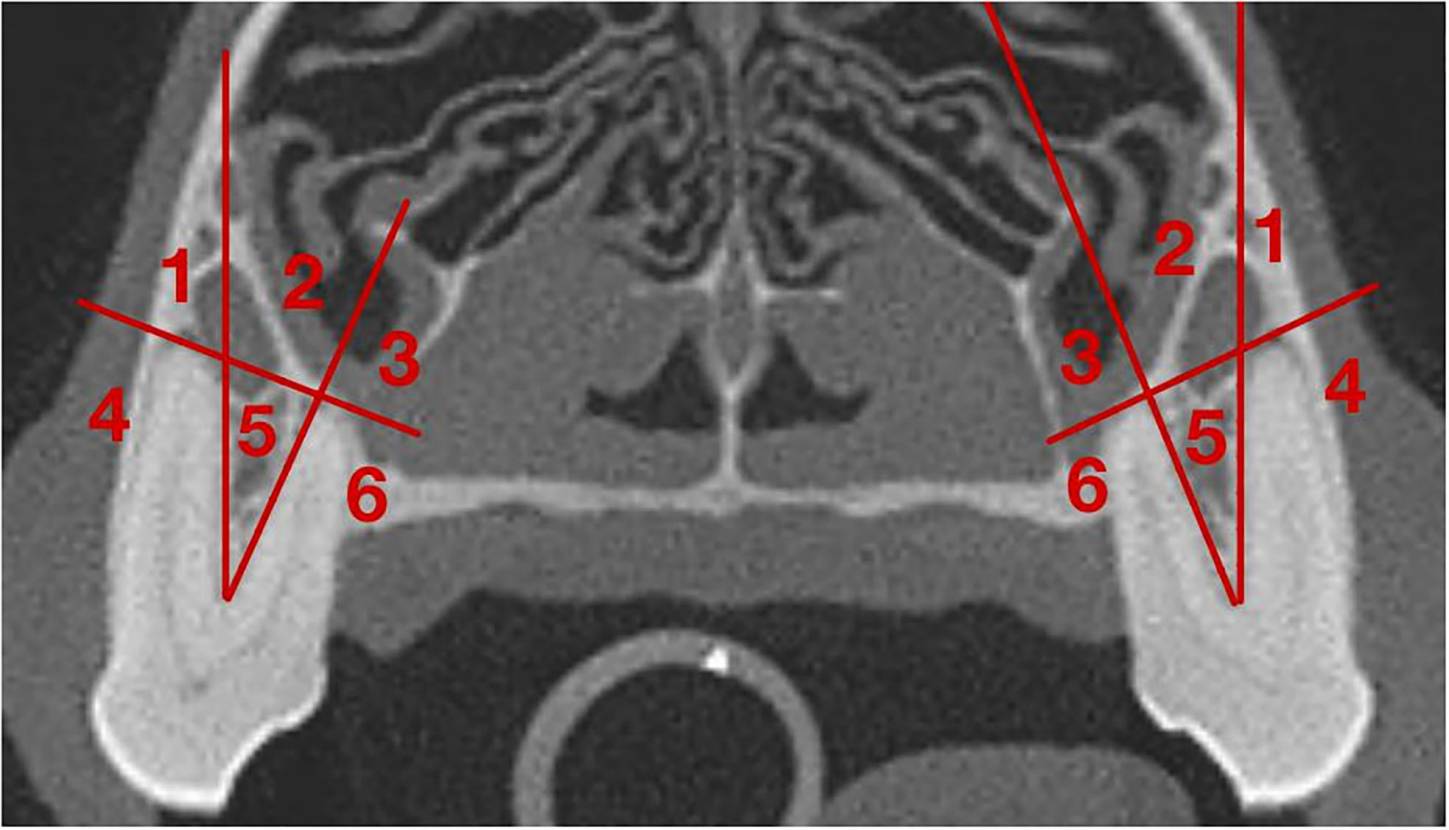
Grid for the IOC relative to the mesial roots of the P4. **(1)** Section 1: Directly Apical to the mesiobuccal root; **(2)** Section 2: Apical to the furcation of the mesial roots; **(3)** Section 3: Directly apical to the mesiopalatal root; **(4)** Section 4: Buccal to the mesiobuccal root; **(5)** Section 5: In the furcation of the mesial roots; **(6)** Section 6: Palatal to the mesiopalatal root.

**Figure 2:**
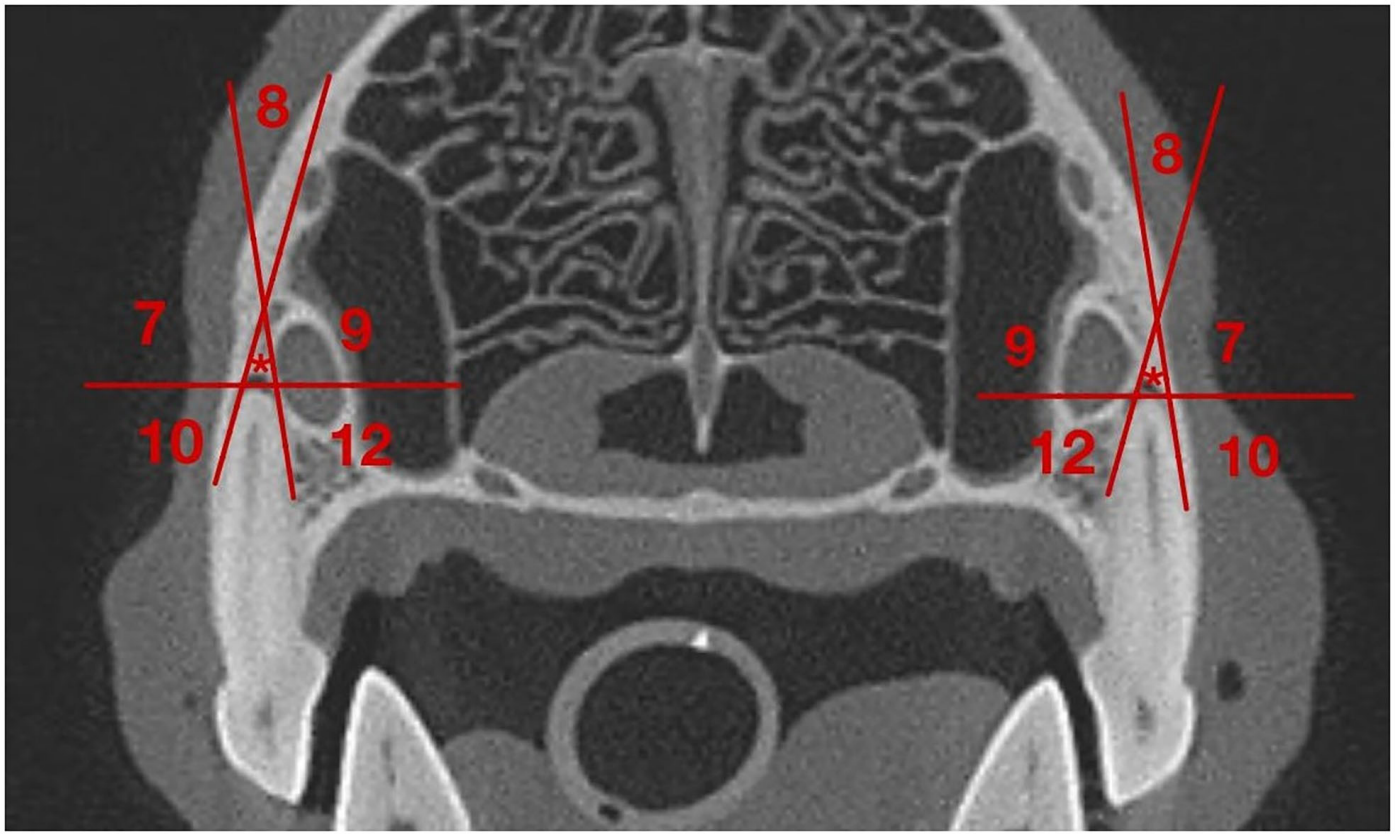
Grid for the IOC relative to the distal root of the P4. **(7)** Section 7: Apical and buccal to the distal root; **(8)** Section 8: Indirectly apical to the distal root; **(9)** Section 9: Apical and palatal to the distal root; **(10)** Section 10: Directly buccal to the distal root, (*) Section 11: Directly apical to the distal root; **(12)** Section 12: Directly palatal to the distal root.

**Figure 3:**
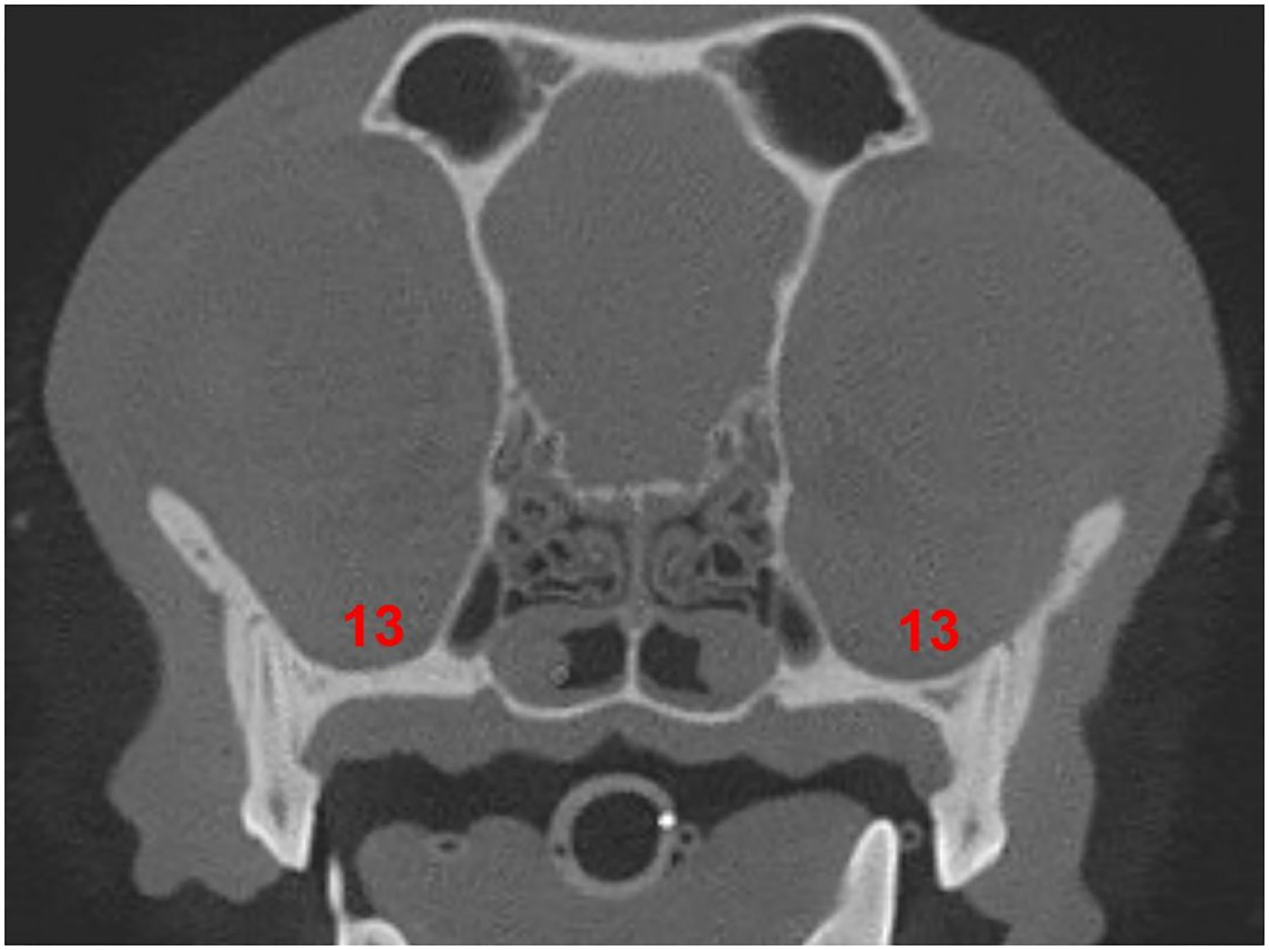
Section 13 relative to the roots of the P4. **(13)** Section 13: caudal to the maxillary foramen/ floor of the orbit.

The transverse plane was used to evaluate the position of the IOC relative to the roots of the left and right P4. This was assessed on the image slice in which the root length appeared at its longest. A grid system designed by the author categorized the position of the IOC relative to the mesial roots and the distal root of the P4. For the grid system, sections 1–6 pertained to the mesial roots (Table 1 and Figure 1), and sections 7–12 pertained to the distal root (Table 1 and Figure 2). Section 13 pertained to the mesial and distal roots (Table 1 and Figure 3). The position of the IOC was found to be either entirely in one section or two or more sections. An additional section, Section 13 (caudal to the maxillary foreman/floor of the orbit), was identified and included as a section relative to the mesial and distal roots of P4. Not all identified positions were present for each skull type.

**Table 1:** Definition of the sections relative to the roots of P4.

**Mesial roots**
Section 1	Directly apical to the mesiobuccal root	
Section 2	Apical to the furcation of the mesial roots	
Section 3	Directly apical to the mesiopalatal root	
Section 4	Buccal to the mesiobuccal root	
Section 5	Furcation of the mesial roots	
Section 6	Palatal to the mesiopalatal root	
**Distal Root**
Section 7	Apical and buccal to the distal root	
Section 8	Indirectly apical to the distal root	
Section 9	Apical and palatal to the distal root	
Section 10	Directly buccal to the distal root	
Section 11	Directly apical to the distal root	
Section 12	Directly palatal to the distal root	
**Mesial and Distal Roots**
Section 13	Caudal to the maxillary foramen/ floor of the orbit	

**Figure 4:**
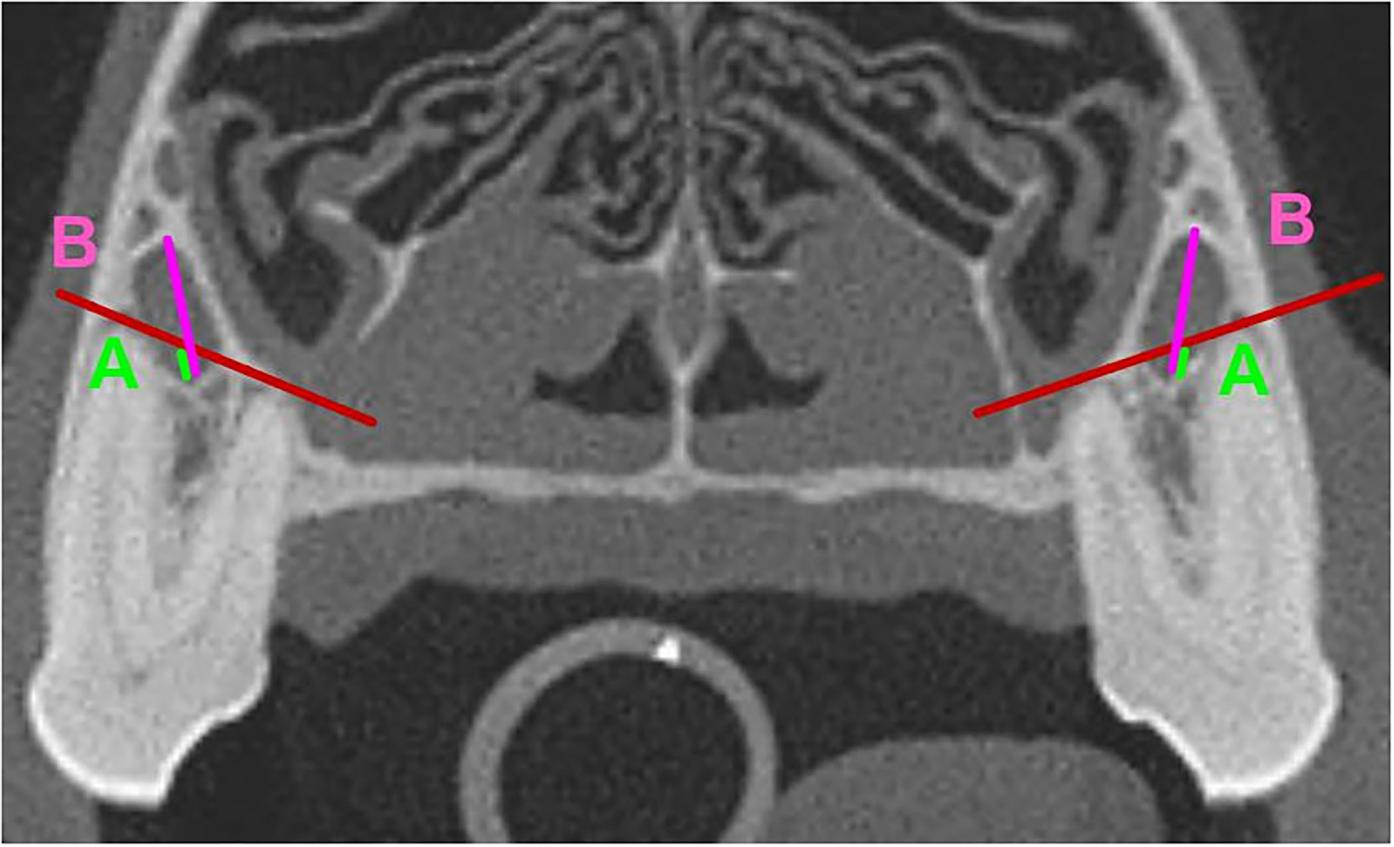
Mesial Roots of the P4. **(A)** (neon Green): Vertical Height of the portion of the IOC in Section 5. **(B)** (fuchsia): Total Vertical height of the IOC. The portion (ratio) of the IOC in Section 5 was calculated as A/B.

**Figure 5:**
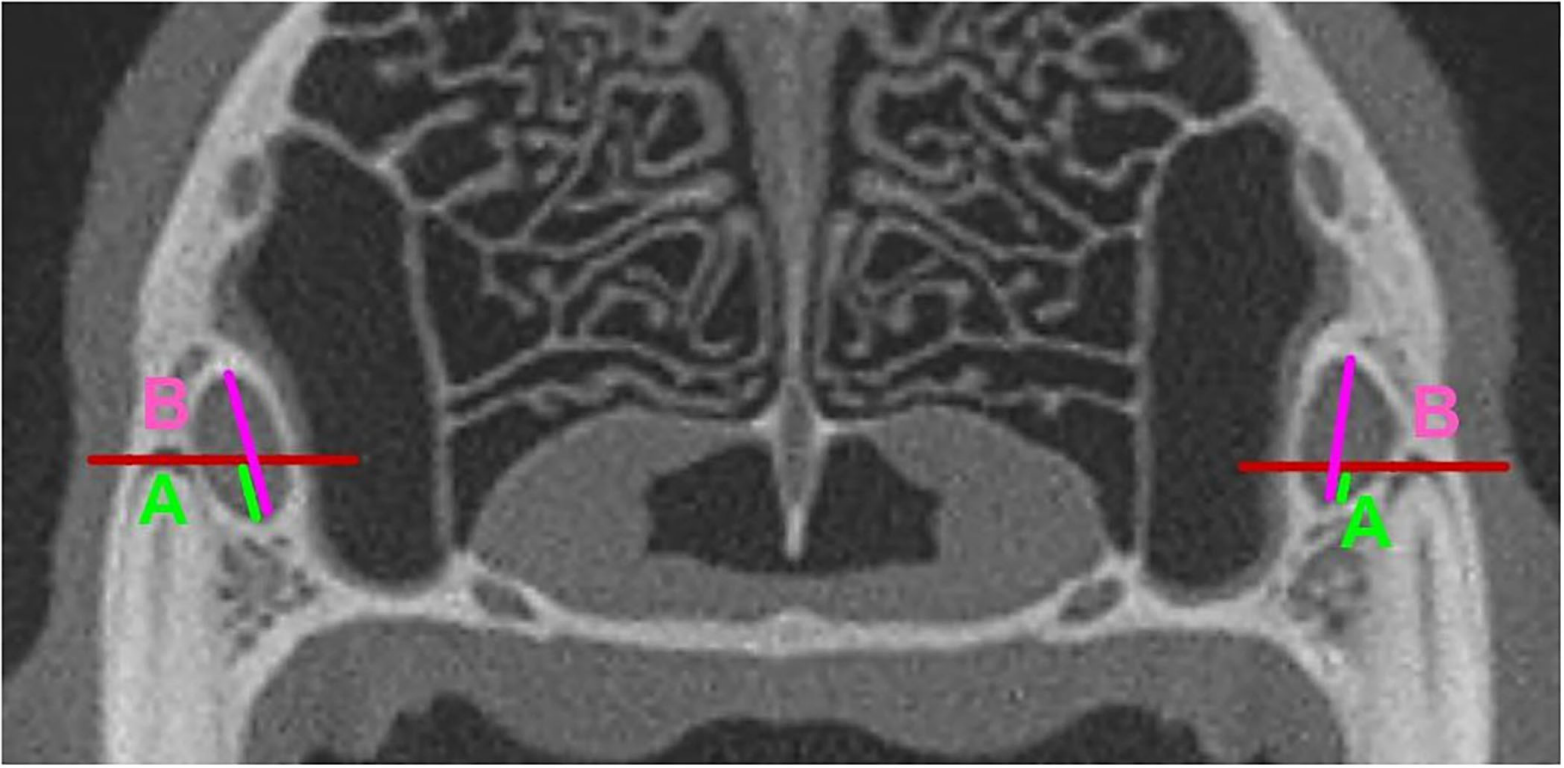
Distal Roots of the P4. **(A)** (neon Green): Vertical Height of the portion of the IOC in Section 12. **(B)** (fuchsia): Total Vertical height of the IOC. The portion (ratio) of the IOC in Section 12 was calculated as A/B.

Of particular clinical interest was the IOC being positioned fully or partially in Section 5 or Section 12, as these would be the most at-risk sections for iatrogenic damage to the IOC during a surgical procedure. To glean more anatomical information at these locations, the ratio of the vertical height of the IOC within these sections to the total vertical height of the IOC was calculated. The calculated ratios were placed into the following groups: Group A: No IOC in the specific Section; Group B: 0 <IOC ≤ 1/3; Group C: 1/3 <IOC ≤ 2/3; Group D: 2/3 <IOC <1; Group E: Entire IOC in the specific Section (Figures 4, 5). Data on the symmetry between the left and right IOC with portions in Section 5 and Section 12 were analyzed for each skull type.

### Statistical analysis

To find a difference of 30% in the proportion of dogs with different skull types having the infraorbital canal located dorsal to the alveoli of the maxillary P4 roots, a total of 40 animals in each group of skull types is required to achieve a statistical power of 80% and a confidence of 95%. STATA 16.1 (StataCorp LLC, TX) was used for the calculations.

The total number and percent of possible sections relative to the mesial and distal roots of both P4 for each dog were calculated for each skull type group and the total population. The age, weight, facial index, and portion of IOC in Section 5 and Section 12 were described using means and standard deviation. The associations between the skull type and the portion of the IOC in Section 5 and Section 12 were evaluated for normality, and if not met, it was analyzed using the Kruskal-Wallis test to compare the three skull type groups. The correlation between weight and the portion of IOC in Section 5 and Section 12 was analyzed using Pearson's correlation coefficient for the three skull type groups. A *p*-value of 0.05 was used to evaluate statistical significance. SAS v9.4 (SAS Institute Inc., Cary, NC) was used for all statistical analyses.

## Results

### Animals

A total of 120 dogs [60 males (51 castrated and 9 sexually intact) and 60 females (55 spayed and 5 sexually intact)] were included in this study. There were 40 dogs for each skull type category: mesocephalic, brachycephalic, and dolichocephalic. The mean ± SD age for all 120 dogs was 6.7 ± 3.53 years (range 1–16 years).

The following breeds were determined to be mesocephalic by their breed and facial index: Labrador retriever (*n*= 9), Australian shepherd (5), Siberian husky (4), basenji (3), Australian cattle dog (2), bichon frise (2), border collie (2), cairn terrier (2), golden retriever (2), Pembroke Welsh corgi (2), and Catahoula leopard dog, German shorthaired pointer, miniature pinscher, Norfolk terrier, silky terrier, vizsla, and West Highland white terrier (1 each). The mean ± SD age of the mesocephalic dogs was 7.10 ± 3.90 years (range, 1–16 years), mean body weight was 20.90 ± 10.71 kg (range 2.64–39.09 kg), and mean facial index was 132.96 ± 16.34 (range 104.12–165.10).

The following breeds were determined to be brachycephalic by their breed and facial index: Chihuahua (*n* = 10), American Staffordshire/Pit Bull Terriers (8), King Charles Spaniel (3), Boston Terrier (3), Shih Tzu (3), and American Bulldog, Boxer, Chow Chow, Mastiff, and French Bulldog (one each); there were also eight mixed-breed dogs (Pit Bull cross (2), Terrier cross (2), Chihuahua cross (1), Pekingese cross (1), Rottweiler cross (1), and Shih Tzu cross (1). The mean ± SD age of the brachycephalic dogs was 6.13 ± 3.59 years (range 1–12 years), mean body weight was 14.71 ± 12.46 kg (range 2.07–54 kg), and mean facial index was 233.66 ± 75.85 (range 154.64–523.80).

The following breeds were determined to be dolichocephalic by their breed and facial index: Dachshund (6), standard Poodle (6), Italian Greyhound (5), Basset Hound (3), Greyhound (3), Belgian Tervuren (2), Doberman Pinscher (2), Welsh Terrier (2), Whippet (2), and Collie, English Setter, Great Dane, and Saluki (1 each); there were three mixed-breed dogs (Doberman Pinscher cross (1), standard Poodle cross (1), and Anatolian Shepherd cross (1). One Borzoi and one rough-coated Collie were determined to be dolichocephalic based on their breed alone because the values required to calculate the facial index could not be determined from the CBCT field of view. They were not included in the calculation for the mean skull index. The mean ± SD age of the dolichocephalic dogs was 6.88 ± 3.09 years (range 1–14 years), mean body weight was 21.27 ± 11.31 kg (range 5–48.59 kg), and mean facial index was 94.28 ± 16.86 (range 66.09–130.54).

### Position of the IOC relative to mesial roots of the P4

**Figure 6:**
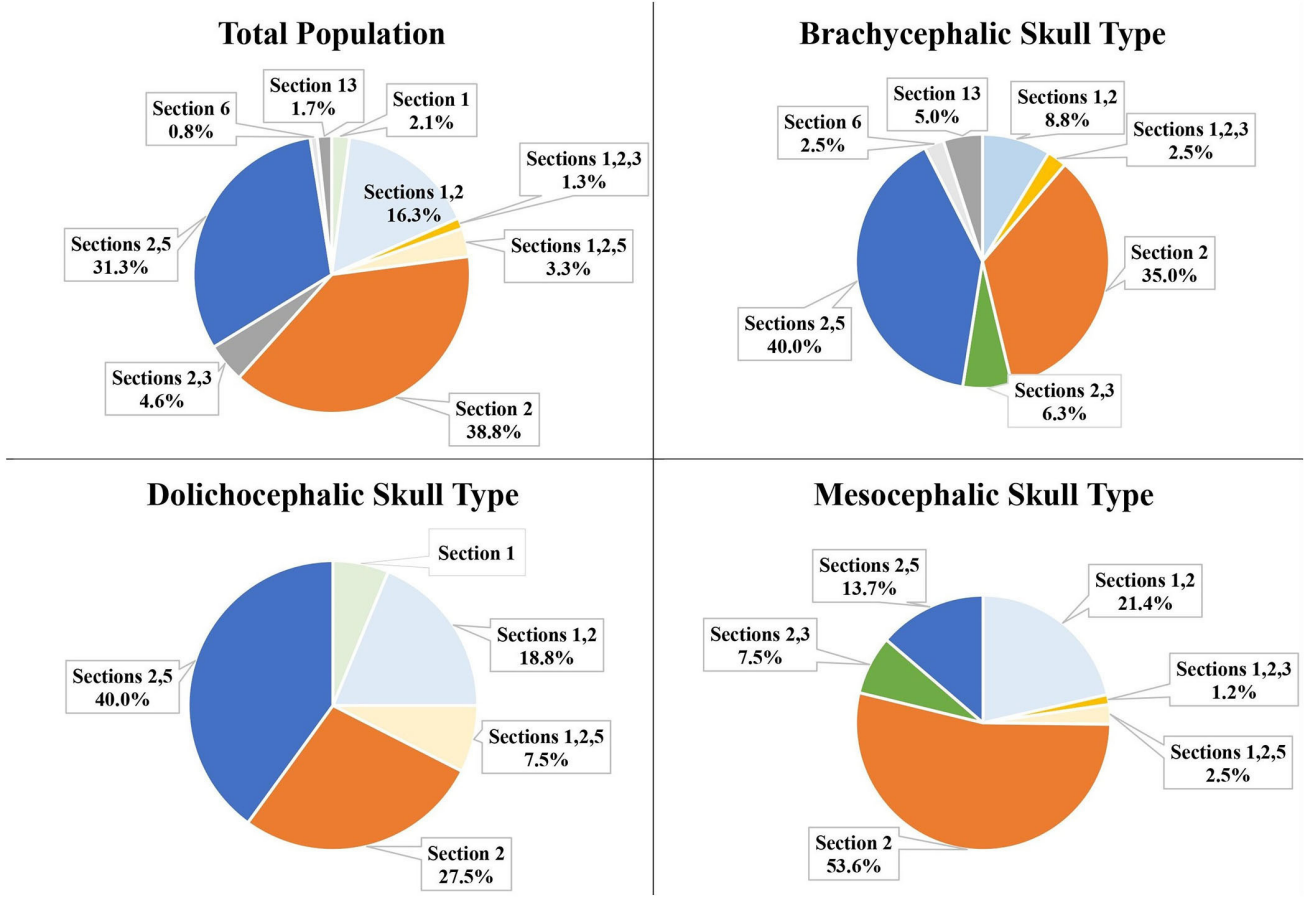
Position of the IOC and Section 13 relative to the mesial roots of the P4 for the total population and the three different skull types.

Among the total population, eight IOC positions were identified relative to the mesial roots of the P4. Section 13 was also identified relative to the mesial roots of P4 (Figure 6).

For the total population, the most frequent position of the IOC was Section 2 (apical to the furcation of the mesial roots) at 38.8%. The second most frequent position was Sections 2 and 5 (partially in the furcation and partially apical to the furcation of the mesial roots of P4) at 31.3% (Figure 6).

The most frequent position of the IOC for the mesocephalic skull type was Section 2 at 53.8%. For the brachycephalic and the dolichocephalic skull types, it was Sections 2 and 5 at 40.0% each (Figure 6).

### Position of the IOC relative to distal root of the P4

**Figure 7:**
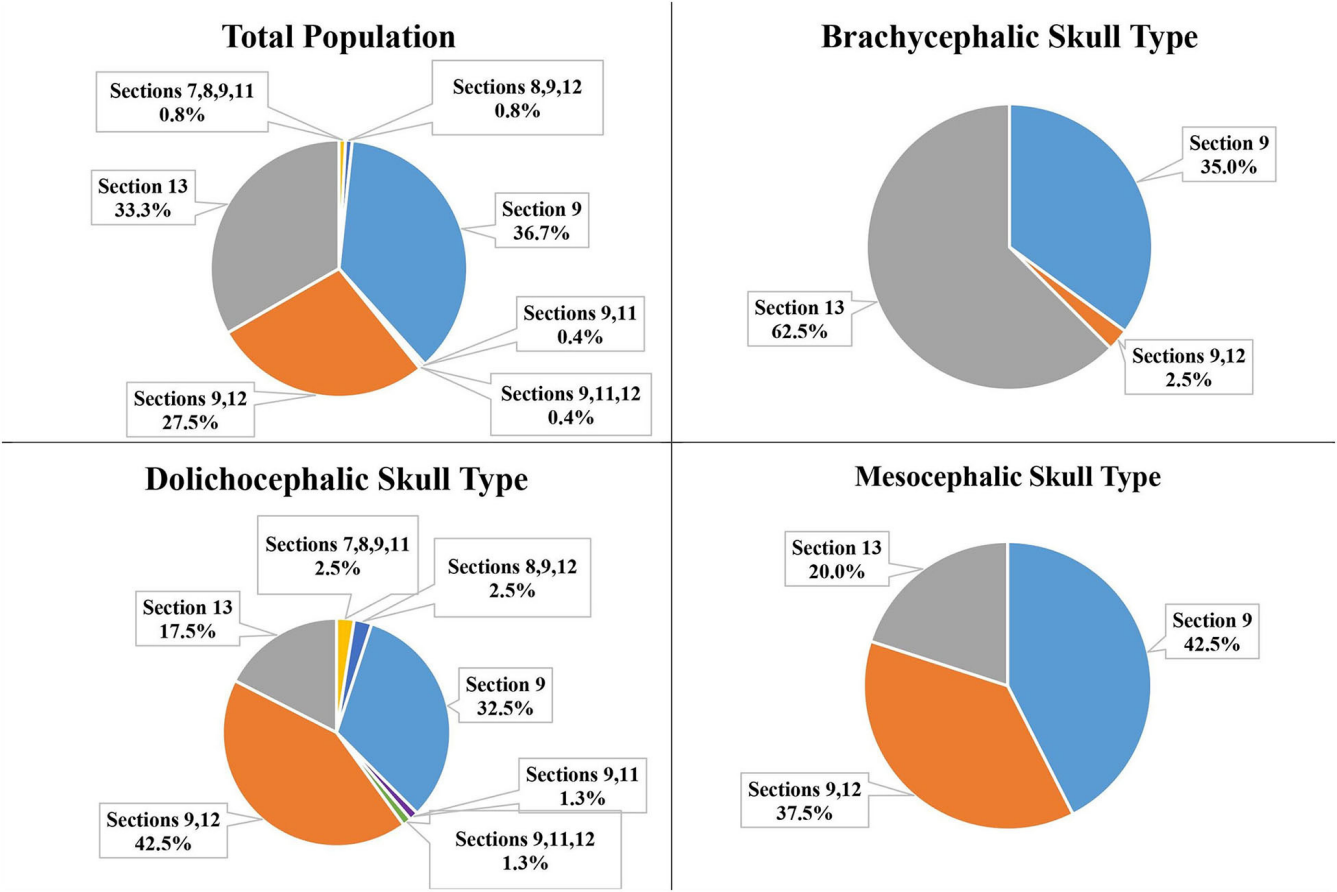
Position of the IOC and Section 13 relative to the distal root of the P4 for the total population and the three different skull types.

For the total population, six IOC positions were identified relative to the distal root of the P4. Section 13 was also identified relative to the distal root of P4 (Figure 7).

Section 9 (apical and palatal to the distal root) was the most frequent position of the IOC for the total population (36.7%), mesocephalic skull type (42.5%), and brachycephalic skull type (35.0%). The dolichocephalic skull type's most frequent position was Sections 9 and 12 (directly palatal and partially apical and palatal to the distal root) at 42.5% (Figure 7).

### IOC in section 5 for mesial roots of the P4

**Figure 8:**
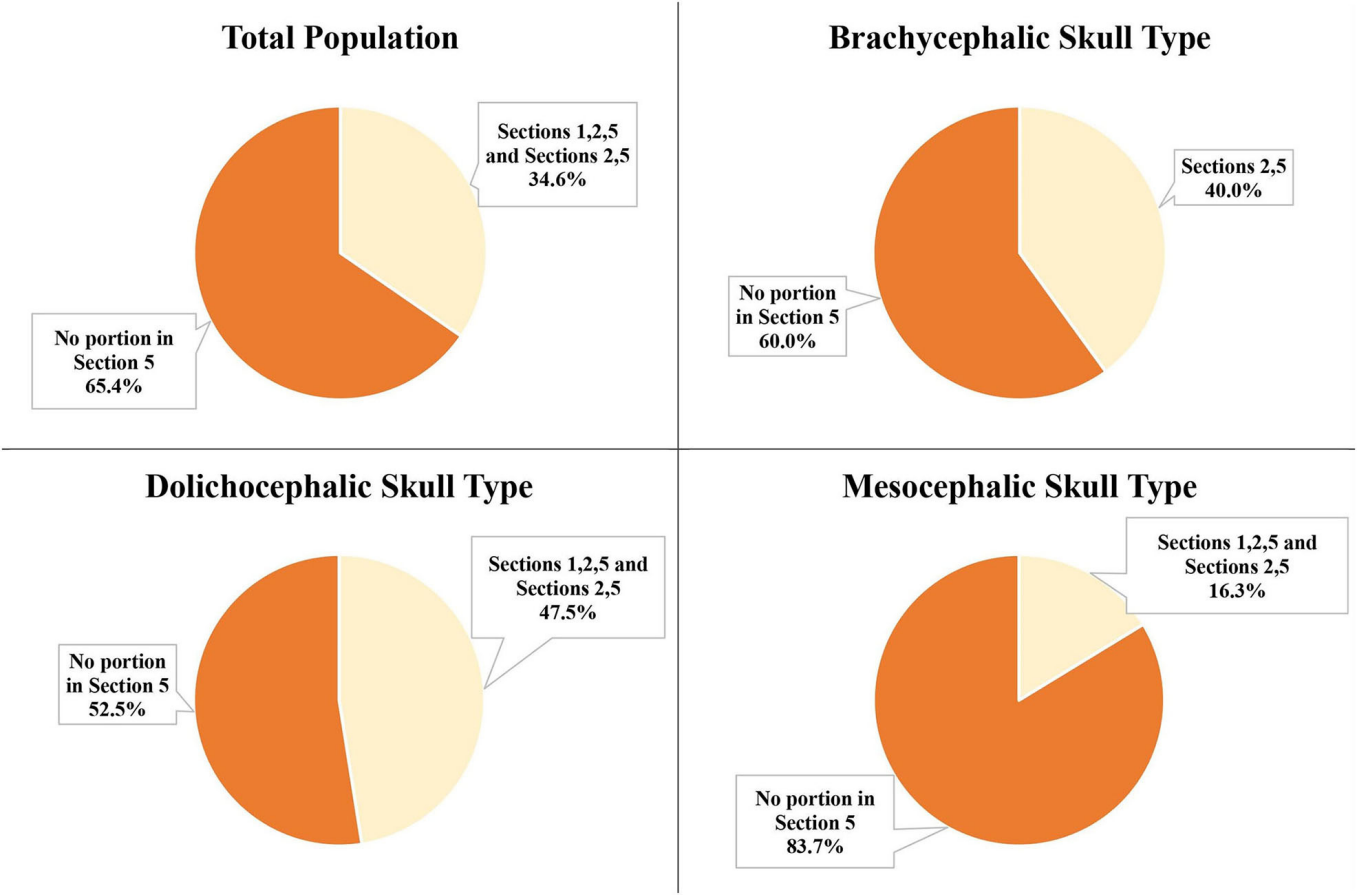
IOC fully or partially in Section 5 at the mesial roots of the P4 for the total population and the three different skull types.

Of the total teeth population, 34.6% had the IOC fully or partially within Section 5 (in the furcation of the mesial root). For the individual skull type groups, it was 40.0% for the brachycephalic skull type, 47.5% for the dolichocephalic skull type, and 16.25% for the mesocephalic skull type (Figure 8). The difference between the skull types was statistically significant (*p*-value = 0.02). The dolichocephalic skull type (*p*-value = 0.05) and the brachycephalic skull type (*p*-value = 0.05) were more likely to have the IOC fully/partially within Section 5 than the mesocephalic skull type.

There was a significant negative correlation between the patient's weight and the portion of the IOC in Section 5 for the mesocephalic skull type (*p*-value = 0.05), the brachycephalic skull type (*p*-value = 0.0015), and the dolichocephalic skull type (*p*-value = 0.0089). As the weight increased, the portion of the IOC in Section 5 decreased.

The left-right symmetry was analyzed in the population of dogs with at least one IOC fully or partially within Section 5. For the brachycephalic (16/16) and dolichocephalic (19/19) skull types, 100% of these individuals are symmetrical for the left and right IOC partially within Section 5. For the mesocephalic skull type, 85.7% (6/7) of the individuals were symmetrical.

### Portion of the IOC in section 5 at mesial roots of the P4

**Figure 9:**
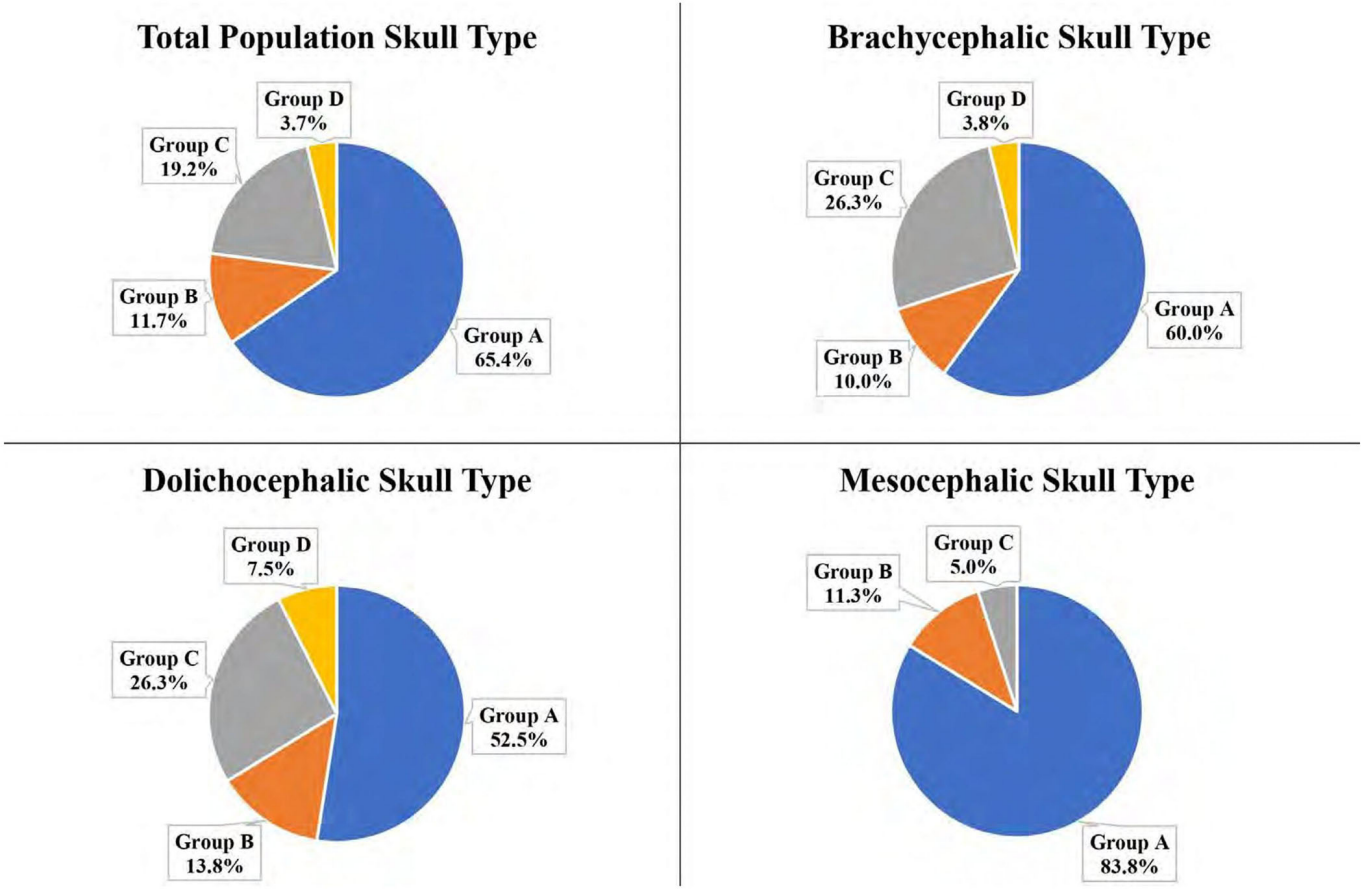
Portion of the IOC within Section 5 at the mesial roots of the P4 for the total population and the three different skull types: Group A: No IOC in Section 5; Group B: 0 <IOC ≤ 1/3; Group C: 1/3 <IOC ≤ 2/3; Group D: 2/3 <IOC <1; Group E: Entire IOC in Section 5. *Groups not pictured had 0%.

The portion of the IOC within Section 5 (furcation between the mesial roots) was calculated and placed into Groups A, B, C, D, or E. No IOC was entirely within Section 5 (Group E) for the total population. The majority of the total population (65.4%) and the individual three skull types were in Group A (no IOC in section 5; Figure 9).

The second most frequent group for the total population (19.2%), the brachycephalic skull type (26.3%), and the dolichocephalic skull type (26.3%) was Group C (1/3 <IOC ≤ 2/3). For the mesocephalic skull type, it was Group B (0 <IOC ≤ 1/3) at 11.3%.

### IOC in section 12 for distal root of the P4

**Figure 10:**
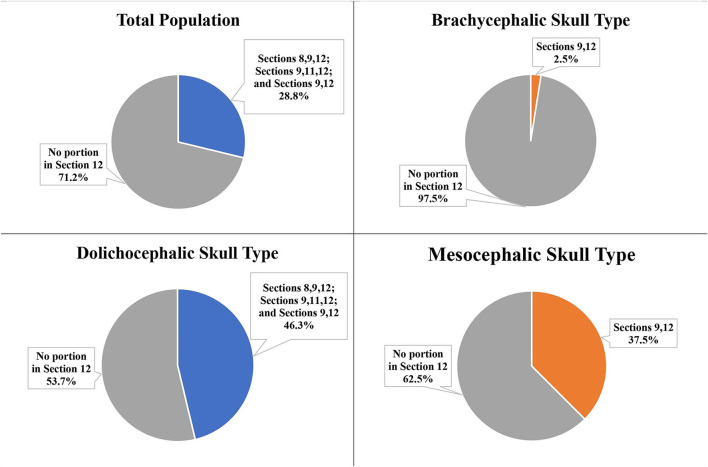
IOC fully or partially in Section 12 at the distal root of the P4 for the total population and the three different skull types.

Of the total teeth population, 28.8% had the IOC fully or partially within Section 12 (directly palatal to the distal root). For the individual skull types, it was 46.3% for the dolichocephalic skull type, 37.5% for the mesocephalic skull type, and 2.5% for the brachycephalic skull type (Figure 10). The dolichocephalic skull type was significantly more likely than the mesocephalic skull type to have the IOC within Section 12 (*p*-value = 0.0282). Only the mesocephalic skull type had a significant negative correlation between the patient's weight and the portion of the IOC in Section 12 (as the weight increased, the portion of the IOC in Section 12 decreased). The brachycephalic skull type data were excluded from this statistical analysis because the sample size was too small.

The left–right symmetry was analyzed in the population of dogs with at least one IOC fully or partially within Section 12. In the brachycephalic skull type group, only one individual met this condition. This individual was symmetrical for the left and right IOC partially within Section 12. The dolichocephalic skull type had 94.7% (18/19), and the mesocephalic skull type had 87.5% (14/16) symmetrical individuals.

### Portion of the IOC in section 12 at distal root of the P4

**Figure 11:**
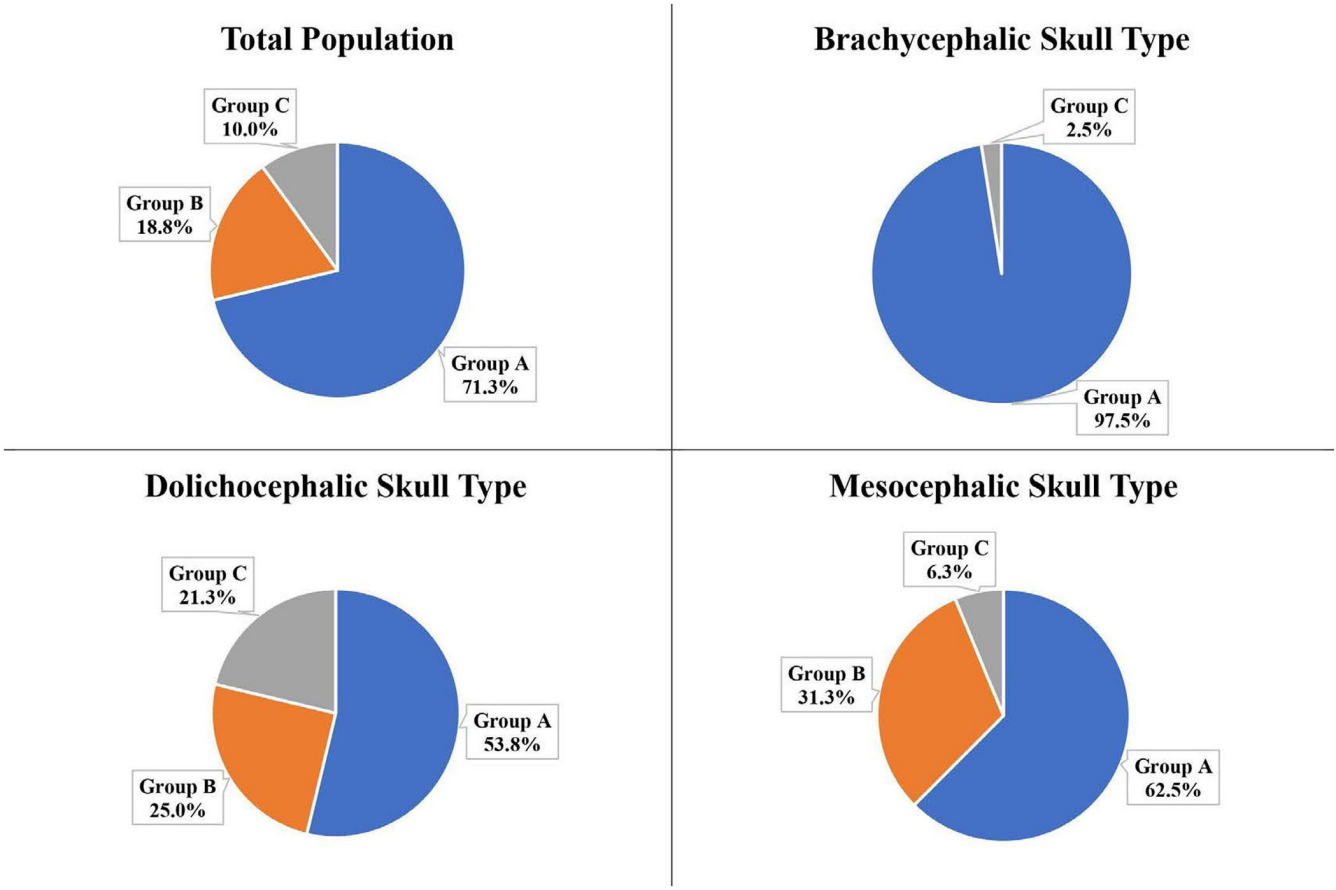
Portion of the IOC within Section 12 at the distal root of the P4 for the total population and the three different skull types: Group A: No IOC in Section 12; Group B: 0 <IOC ≤ 1/3; Group C: 1/3 <IOC ≤ 2/3; Group D: 2/3 <IOC <1; Group E: Entire IOC in Section 12. *Groups not pictured had 0%.

The portion of the IOC within Section 12 (directly palatal to the distal root) was calculated and placed into Groups A, B, C, D, or E. For the total population, no IOC was in Group D (2/3 <IOC <1) or Group E (entire IOC in Section 12). The majority of the total population (71.3%) and the three skull types had the IOC in Group A (no IOC in Section 12; Figure 11).

The second most frequent group for the total population (18.8%), the mesocephalic skull type (31.3%), and the dolichocephalic skull type (25.0%) was Group B (0 <IOC ≤ 1/3). The second most frequent group for the brachycephalic skull type was Group C (1/3 <IOC ≤ 2/3) at 2.5% (Figure 11).

### Section 13 at mesial roots and distal root of the P4

**Figure 12:**
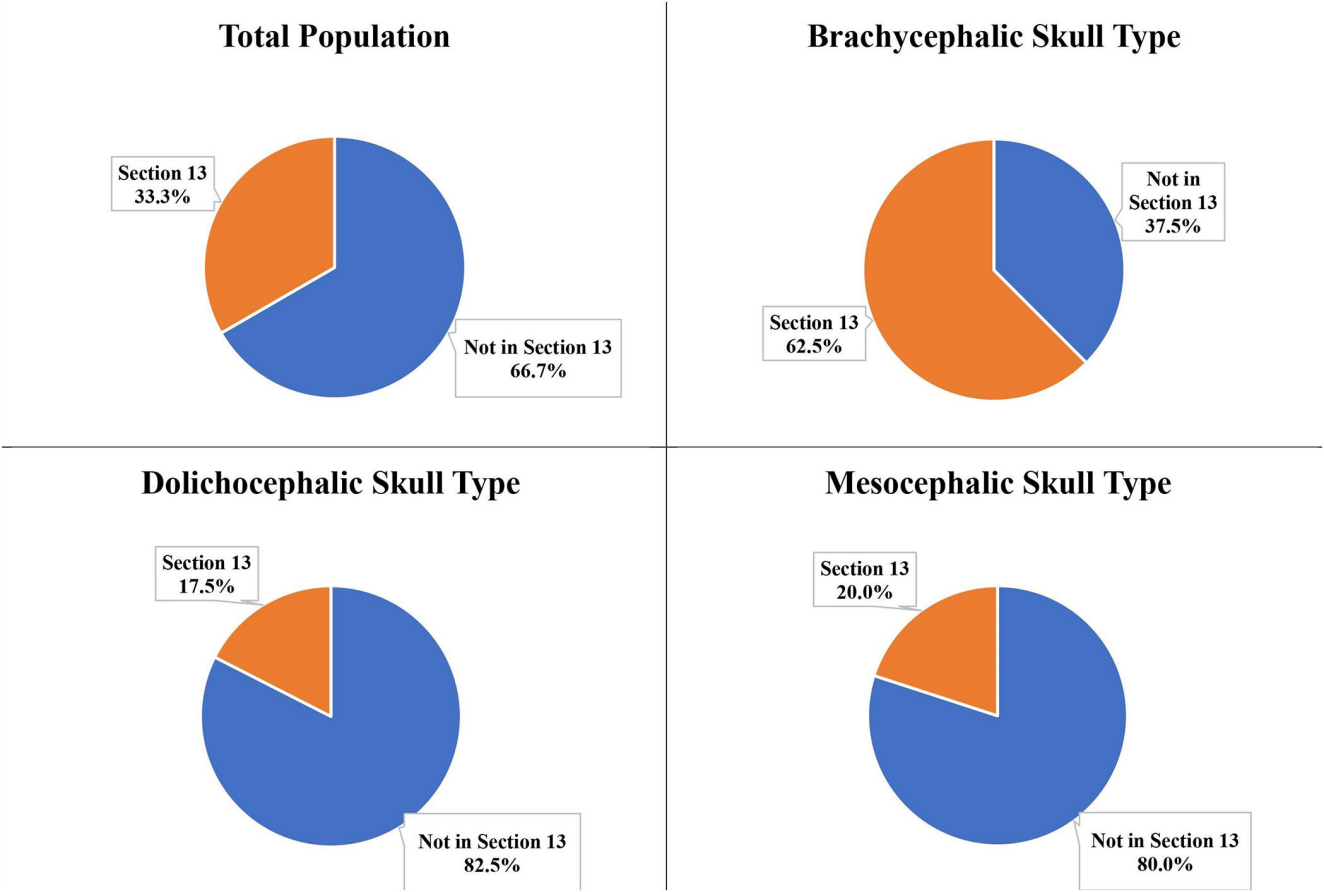
Section 13 relative to the distal roots of the P4 for the total population and the three different skull types.

Of the total population, only two dogs (both brachycephalic skull type) had all three roots of the P4 in Section 13 (caudal to the maxillary foramen/ floor of the orbit). Neither the mesocephalic nor the dolichocephalic skull types had any dogs with Section 13 present at the level of the mesial roots of P4. Section 13 was present at the level of the distal root of the P4 for 33.3% of the total population, 62.5% of the brachycephalic skull type, 20.0% of the mesocephalic skull type, and 17.5% of the dolichocephalic skull type (Figure 12).

## Discussion

In this study, a CBCT was used to determine the position of the IOC relative to the roots of the P4 for the three canine skull types. This study reported that the most frequent position of the IOC relative to the mesial roots of the P4 for the total population was Section 2 (apical to the furcation of the mesial roots) at 38.8%. This result agreed with sources that stated the anatomic position of the IOC was dorsal to the mesial roots of the P4 (**9,13**). This study specifically identified that the position was dorsal to the furcation of the mesial roots and not directly dorsal to the mesial roots.

The most frequent position of the IOC relative to the mesial roots of the P4 varied among the individual skull types. Section 2 was the most frequent location for the mesocephalic skull type (53.8%), coinciding with the total population results. However, for the dolichocephalic skull type (40.0%) and brachycephalic skull type (40.0%), it was Sections 2 and 5 (partially in the furcation and partially apical to the furcation of the mesial roots). The position variations documented in this study between the skull types support the variations reported in the literature for the IOC relative to the mesial roots of the P4.

For the dolichocephalic skull type, a previous report indicated that the IOC was positioned immediately dorsal to the apex of the mesiobuccal root of the P4 (13). The current study confirmed that this position was a possibility for this skull type, but it was not the most frequent position. The previous report used one dolichocephalic skull-type cadaver to determine the position of the IOC. Assessment of a single skull does not account for possible variations within an individual population.

The IOC positioned fully or partially in Section 5 (in the furcation of the mesial roots) of P4 was of interest to the author because of its significance during surgical extraction and surgical endodontics involving this tooth. The descriptive data indicated that the dolichocephalic and brachycephalic skull types tended to have a higher number of IOC partially positioned within Section 5, which was significant when compared to the mesocephalic skull type. In this study's population, no skull type had the IOC entirely in Section 5. This was important to note because of the concern for trauma to the neurovascular bundle when performing an alveolectomy of alveolar bone on the buccal aspect of the mesiopalatal root of the P4.

Regarding the position of the IOC relative to the distal root of the P4, the total population's most frequent position was Section 9 (apical and palatal to the distal root) at 36.7%. This indicated that the IOC was more often located apical and palatal to the root and not directly apical to this root.

The most frequent position of the IOC relative to the distal root of the P4 differs between the skull types. Sections 9 and 12 (partially apical and palatal as well as directly palatal to the distal root) were the most frequent position for the dolichocephalic skull type at 42.5%. Section 9 (apical and palatal to the distal root) was the most frequent position for the mesocephalic (42.5%) and the brachycephalic skull types (35.0%). However, when observing all the possible sections relative to the distal root, Section 13 (caudal to the maxillary foramen/ floor of the orbit) was the most frequent section for the brachycephalic skull type at 62.5%. Because of this high percentage, extra precaution was taken with surgical procedures involving the distal root of the P4 to avoid iatrogenic trauma to the eye in the brachycephalic skull types (8).

The IOC positioned fully or partially in Section 12 (directly palatal to the distal root) were highlighted because of their significance during surgical procedures involving this root. The brachycephalic skull type was excluded from this data set because of the small number of IOC positioned in Section 12. Once the brachycephalic skull type was excluded, the dolichocephalic skull type was more likely to have a portion of the IOC positioned in Section 12 than the mesocephalic skull type. This may indicate that extra precaution may be required during surgical procedures on this root in the dolichocephalic skull type.

In this study, the mesocephalic skull type results mirrored the results for the total population. This may justify using the mesocephalic skull type in research studies involving the IOC and P4.

The weights of the dogs were known and analyzed to determine if there was a correlation between weight and portion of the IOC in either Section 5 or Section 12. At the mesial roots of P4, there was a significant negative correlation between weight and the portion of the IOC in Section 5 for all three skull types. At the distal root of the P4, there was a significant negative correlation between weight and the portion of the IOC in Section 12 for only the mesocephalic skull type. For the mesocephalic skull type, this study indicated that as the weight increased, the portion of the IOC in both Section 5 and Section 12 decreased. However, these data may be biased based on the weight distribution between the skull types, as weight was not an exclusion parameter.

When classifying a small breed dog to be less than 10 kg, a medium breed dog to be 10–25 kg, and a large breed dog to be greater than 25 kg, the mesocephalic and the dolichocephalic skull types were proportional to the weight distribution between these classes. However, the brachycephalic skull types were disproportional between the weight class distribution compared to the other two skull types. Over half (21/40) of the brachycephalic skull type population were less than 10 kg. Previously published studies have analyzed the mandibular first molar roots regarding their location relative to the mandibular canal (12, 14). The most recent article reported that the patient's size significantly correlated with the position of the roots of the mandibular first molar being buccally, lingually, or dorsally located (14). That study eliminated bias by defining the weight classification for extra-small, small, medium, and large breed dogs, and kept the distribution of weight equal between the different skull types. A future study on the position of the IOC relative to the P4 could eliminate this bias by evenly distributing weight classes between the individual skull type groups.

Another potential bias in the data was the overlapping values for the facial index between the different skull types. A previous study analyzed the mandibular first molar roots relative to the mandibular canal using only the mesocephalic skull type (12). That study used the facial index range of 96–163 to determine if the cadaver skulls (breeds unknown) were mesocephalic. In this study, most breeds were known and were used to determine the skull type group. At the same time, their facial index was calculated but not used to categorize them unless it was a mixed-breed dog: mesocephalic skull type was 104–165, dolichocephalic skull type was less than 104, and brachycephalic skull type was greater than 165. Because the breed determined the skull type group, the brachycephalic population had four individuals with facial index values overlapping with the mesocephalic skull type values, the mesocephalic population had five individuals overlapping with the brachycephalic skull type values, and the dolichocephalic population had nine individuals overlapping with the mesocephalic skull type values. None of the individuals with overlapping facial index values were considered mixed-breed. The breeds of these overlapping individuals were commonly accepted within their respective skull type groups. Different phenotypes seen within an individual breed could explain this overlap, but demonstrating this theory goes beyond the scope of this study. The study could have selected one specific breed using one weight class for each of the three skull types to correct this bias.

Due to the limitation of the CBCT software used in the study, the exact areas and volumes of the IOC could not be calculated. It may have been beneficial and more accurate to use third-party software to obtain measurements to determine the exact portion of the IOC within Section 5 and 12.

## Conclusion

Previous literature indicated variations in the position of the IOC relative to the roots of the P4. This study revealed the most frequent position of the IOC relative to the roots of the P4 for the three different skull types. Based on the results, extra precaution should be taken for both the dolichocephalic and the brachycephalic skull types when performing extractions and surgical endodontics on the P4 as there may be an increased risk of iatrogenic trauma to the IOC and the anatomic structures caudal to the IOC.

## Data availability statement

The original contributions presented in the study are included in the article/Supplementary material, further inquiries can be directed to the corresponding author.

## Ethics statement

Ethical review and approval was not required for the animal study because it was retrospective study. Prior to treatment, informed consent for the use of the clinical data was obtained by the owners as part of the practice's intake consent form. Written informed consent for participation was not obtained from the owners because this was a retrospective review of the patient medical record and imaging only.

## Author contributions

ML and KB: project conception and design. SR: statistical analysis. ML: initial draft of the manuscript. All authors contributed to the article and approved the submitted version.

## Conflict of interest

Authors ML and KB are employed by Veterinary Dentistry and Oral Surgery of New Mexico. The remaining author declares that the research was conducted in the absence of any commercial or financial relationships that could be construed as a potential conflict of interest.

## Publisher's note

All claims expressed in this article are solely those of the authors and do not necessarily represent those of their affiliated organizations, or those of the publisher, the editors and the reviewers. Any product that may be evaluated in this article, or claim that may be made by its manufacturer, is not guaranteed or endorsed by the publisher.
